# Everglades virus evolution: Genome sequence analysis of the envelope 1 protein reveals recent mutation and divergence in South Florida wetlands

**DOI:** 10.1093/ve/veac111

**Published:** 2022-12-14

**Authors:** Monica C Valente, Dhani Prakoso, Amy Y Vittor, Erik M Blosser, Nabil Abid, Ruiyu Pu, Sarah E Beachboard, Maureen T Long, Nathan D Burkett-Cadena, Carla N Mavian

**Affiliations:** Department of Comparative Diagnostic and Population Medicine, College of Veterinary Medicine, University of Florida, Gainesville, FL 32601, USA; Department of Comparative Diagnostic and Population Medicine, College of Veterinary Medicine, University of Florida, Gainesville, FL 32601, USA; Department of Pathology, Emerging Pathogens Institute, College of Medicine, University of Florida, Gainesville, FL 32601, USA; Division of Infectious Diseases and Global Medicine, Department of Medicine, College of Medicine, University of Florida, Gainesville, FL 32601, USA; Florida Medical Entomology Laboratory, Entomology and Nematology Department, University of Florida, Vero Beach, FL 32960, USA; High Institute of Biotechnology of Monastir and Laboratory of Transmissible Diseases and Biological Active Substances, Faculty of Pharmacy of Monastir, University of Monastir, Monastir 5089, Tunisia; Department of Comparative Diagnostic and Population Medicine, College of Veterinary Medicine, University of Florida, Gainesville, FL 32601, USA; Department of Pathology, Emerging Pathogens Institute, College of Medicine, University of Florida, Gainesville, FL 32601, USA; Department of Comparative Diagnostic and Population Medicine, College of Veterinary Medicine, University of Florida, Gainesville, FL 32601, USA; Florida Medical Entomology Laboratory, Entomology and Nematology Department, University of Florida, Vero Beach, FL 32960, USA; Department of Pathology, Emerging Pathogens Institute, College of Medicine, University of Florida, Gainesville, FL 32601, USA; Department of Pathology, College of Medicine, University of Florida, Gainesville, FL 32601, USA

**Keywords:** Everglades virus, *Culex cedecei*, evolution, phylogeography, phylogenetics

## Abstract

Everglades virus (EVEV) is a subtype (II) of Venezuelan equine encephalitis virus (VEEV), endemic in southern Florida, USA. EVEV has caused clinical encephalitis in humans, and antibodies have been found in a variety of wild and domesticated mammals. Over 29,000 *Culex cedecei* females, the main vector of EVEV, were collected in 2017 from Big Cypress and Fakahatchee Strand Preserves in Florida and pool-screened for the presence of EVEV using reverse transcription real-time polymerase chain reaction. The entire 1 *E1* protein gene was successfully sequenced from fifteen positive pools. Phylogenetic analysis showed that isolates clustered, based on the location of sampling, into two monophyletic clades that diverged in 2009. Structural analyses revealed two mutations of interest, A116V and H441R, which were shared among all isolates obtained after its first isolation of EVEV in 1963, possibly reflecting adaptation to a new host. Alterations of the Everglades ecosystem may have contributed to the evolution of EVEV and its geographic compartmentalization. This is the first report that shows in detail the evolution of EVEV in South Florida. This zoonotic pathogen warrants inclusion into routine surveillance given the high natural infection rate in the vectors. Invasive species, increasing urbanization, the Everglades restoration, and modifications to the ecosystem due to climate change and habitat fragmentation in South Florida may increase rates of EVEV spillover to the human population.

## Introduction

Everglades virus (EVEV) is subtype II of Venezuelan equine encephalitis virus (VEEV), endemic to southern Florida, USA, which can cause clinical encephalitis in humans (Chamberlain et al. 1964). Early studies demonstrated that EVEV is serologically distinct from the other four subtypes of VEEV, leading to its classification as a unique subtype. This virus has been isolated from mosquitoes and wild rodents from southern Florida, in particular the hardwood hammocks of the Everglades and Big Cypress ecosystems (Chamberlain et al. 1964, 1969; Young, Johnson, and Gauld 1969). The only confirmed vector is the mosquito *Culex cedecei* (Chamberlain et al. 1969; Weaver et al. 1986; Coffey and Weaver 2005), the sole member of the tropical Spissipes section of *Culex* (*Melanoconion*) that is native to the USA. Vector competence studies in other species are limited, but one study demonstrated little competence of *C. nigripalpus* and *Ochlerotatus taeniorhynchus* (Coffey and Weaver 2005). The hispid cotton rat (*Sigmodon hispidus*) and the cotton mouse (*Peromyscus gossypinus*) are considered primary reservoir hosts of EVEV (Chamberlain et al. 1964, 1969; Coffey et al. 2004). As a subtype II virus, EVEV shares a common ancestry with IAB, IC, and ID viruses. EVEV is most closely related to subtype ID, an enzootic strain of VEEV that occurs in Panama, Colombia, Peru, and Venezuela (Ventura, Buff, and Ehrenkranz 1974). EVEV arose approximately 100–150 years ago from a common ID-like ancestor that colonized Florida (Weaver, Bellew, and Rico-Hesse 1992). The mutation of subtypes IAB and ICE leads to the emergence of epizootics of VEEV in Central and South America (Weaver, Bellew, and Rico-Hesse 1992). The potential for EVEV to give rise to epizootic activity, similar to these subtypes, is unknown; restricted vector competence to essentially one species of mosquito, a limited host range, and stringent geographical and ecological boundaries have likely limited epizootic emergence thus far. These impediments could be reduced by the recent introduction of *Culex panocossa*, a competent vector of Panamanian subtype D VEEV (Blosser and Burkett-Cadena 2017). The establishment of a vector of epizootic subtypes could enable EVEV to emerge from its enzootic cycle (Blosser and Burkett-Cadena 2017). Thus, understanding the phylogenetic relationships of EVEV strains in Florida is important. While new knowledge has been gained from understanding the effect of mammalian diversity on transmission dynamics of VEEV, there are limited genetic data for EVEV and, consequently, a gap exists in our knowledge of the evolution of this virus. As of this time, only two whole VEEV genomes are published (Genbank IDs KR260733 and AF075251) (Chamberlain et al. 1964, 1969; Kinney et al. 1998; Forrester et al. 2017).

Clinical human infection has been documented beginning with the documentation of widespread seroconversion of the Seminole Indian tribe living in and around the Florida Everglades subsequent to its discovery in the early 1960s (Work 1964; Ventura, Buff, and Ehrenkranz 1974). Dogs were proposed by the military as sentinels for the emergence of EVEV based on serosurveys conducted in 1975 (Nichols et al. 1975). A more recent study performed on the archived serum of shelter dogs detected seropositive animals from several central and northern counties in Florida (Coffey et al. 2006). Historically, the virus has maintained an ecologically and host-restricted range, with little evidence of additional reservoirs in non-rodent hosts. However, given the mutation of other VEEV subtypes into epizootic or epidemic strains under appropriate climatic and ecological conditions, the probability of the emergence of EVEV as a cause of encephalitis in humans and animals may be increased with the expansion of human habitation and the reduction of in host biodiversity due to the invasion of foreign vertebrate predators, such as the Burmese python (*Python bivittatus*), into the Everglades.

**Figure 1.**
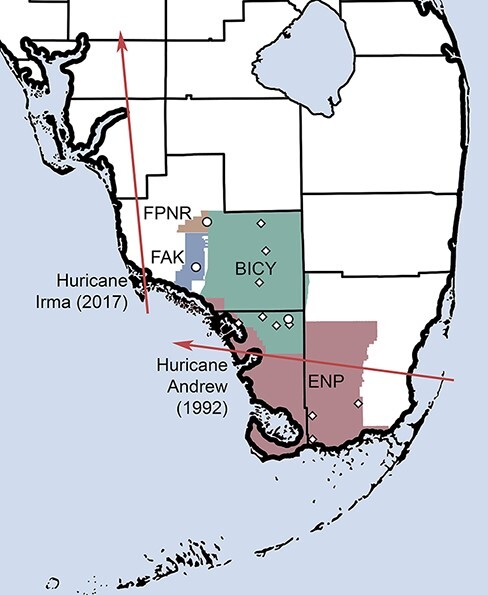
Map of southern Florida showing locations of EVEV samples from public lands. Circles indicate the current study, and diamonds indicate previous studies. Public lands include Florida Panther National Wildlife Refuge (FPNR), Fakahatchee Strand State Park Preserve (FAK), Big Cypress Preserve (BICY), and Everglades National Park (ENP). Arrows indicate approximate hurricane paths of Hurricane Andrew in 1992 and Hurricane Irma in 2017, both affecting South Florida.

EVEV occurs throughout much of the southern Florida peninsula and has been detected in vectors and hosts in the diverse wetland ecosystems of the Everglades that are interspersed among agricultural and developed portions of the region (Coffey et al. 2006; Hoyer et al. 2019; Fish et al. 2021). The Florida Everglades itself is a heterogeneous landscape varying from pine rockland forests to tropical hardwood hammocks linked through a complex water system of swamps, marshes, ponds, and sloughs. Big Cypress (Fig. 1) is a large neighboring subregion that includes several southwestern counties with the main preserve bordering the western boundary of the Everglades National Park. The habitats of Big Cypress consist of cypress swamps, freshwater swamps, sloughs, strands, and prairies extending to saltwater marshes and mangroves along the western and southern edges. These ecosystems are linked by water and have undergone profound shifts in both animal and plant diversity due to anthropogenic activities including tourism, mining, canalization, agriculture, and establishment of invasive plants and animals (Wu, Sklar, and Rutchey 1997; Wu, Wang, and Rutchey 2006). The major long-term effect has been habitat loss and fragmentation. The Fakahatchee Strand Preserve State Park is located due west of Big Cypress (Fig. 1), dominated by a single ‘strand’ swamp, a type of forested wetland composed of a linear shallow freshwater channel in regions where the lack of slope prevents stream formation. EVEV has been isolated from mosquitoes in numerous locations in Big Cypress and Fakahatchee Strand (Fish et al. 2021).

In previous work, we investigated the indirect impacts of the invasion of the Burmese python in South Florida and showed a profound decrease in mammalian diversity, which was associated with a shift in viral kinetics in the vector mosquito (Burkett-Cadena et al. 2021). The loss of non-rodent animals in the Big Cypress Preserve resulted in increased feeding upon cotton rats by *C. cedecei* and a concomitant increase in the overall EVEV infection rate.

Here, we sequenced the E1 protein of EVEV detected in pools of *C. cedecei* trapped during this previous study from two distinct wetlands in South Florida and performed genetic, phylogenetic, and structural analyses to understand the evolution and spread of EVEV across the Everglades.

## Methods

### Mosquito specimen collection

Mosquitoes were collected from September to October 2017 using carbon dioxide–baited Centers for Disease Control and Prevention miniature light traps and resting shelters. Trapping occurred in twelve sites from three ecologically distinct ecosystems within the Greater Big Cypress region, including Big Cypress National Preserve, Fakahatchee Strand Preserve State Park, and Florida Panther National Wildlife Refuge (Fig. 1). However, due to Hurricane Irma, Big Cypress (four sites) and Fakahatchee (two sites) were only accessible during this time period (Fig. 1). Morphological methods were implemented to identify mosquitoes by species. Specimens of *C. cedecei* were pooled into 583 sample groups of 5–25 (mean = 24) adult females according to the collection site and date (Burkett-Cadena et al. 2021). This study involved the collection of mosquitoes from the Everglades. The field permits are number 41545-2017-4R (an institution that granted permission: Florida Panther National Wildlife Refuge), number BICY-2017-SCI-0012 (Big Cypress Preserve), and number 08221714A (Fakahatchee Strand Preserve State Park).

### RNA extraction and reverse transcription real-time polymerase chain reaction

Pooled samples of *C. cedecei* specimens were lysed mechanically (TissueLyser LT, Qiagen, Hilden, Germany) using 5-mm stainless steel beads at 50 oscillations/s for 4 min in 500 μl of 1× phosphate buffered saline. Viral RNA was then extracted and purified using a commercial kit (QIAamp Viral RNA Mini Kit, Qiagen, Hilden, Germany) following the manufacturer’s protocol. Real-Time reverse transcription polymerase chain reaction (PCR) was performed to identify samples positive for alphaviral RNA using a probe and primers of a pan-alphavirus assay as previously described (Giry et al. 2017; Burkett-Cadena et al. 2021). The assay was performed using 4 μl of previously extracted template RNA, 625 nM of each primer F2A, R2A, R3A and R4A, 500 nM of P2 probe, 5 μl of 1-step master mix (TaqMan^®^ Fast Virus 1-Step Mastermix, Applied Biosystems, Foster City, CA), in 20 µl reactions. PCR was performed (QuantStudio 3 Real-Time PCR System, Applied Biosystems, Foster City, CA) with cycling conditions at 50°C for 5 min, 98°C for 20 s, and forty-five cycles consisting of two steps, 98°C for 3 s, and 58°C for 45 s with fluorescence reading using the FAM channel for detection of probe hydrolysis. Positive samples were defined by a cycle threshold of 35 or lower. Processing of mosquito samples prior to viral inactivation was performed in a BSL-3 laboratory to mitigate risk to personnel from unidentified pathogens potentially present in the environmentally sourced mosquitoes.

### Sequencing of alphavirus-positive samples

All positive samples were sequenced using overlapping primers that spanned the gene of the E1 protein (Table 1), and primers were designed for the amplification of four contiguous segments using a sequence (accession number AF075251) retrieved from an online database (GenBank, NCBI-NIH, Bethesda, MD) and Primer3Plus (https://primer3plus.com/cgi-bin/dev/primer3plus.cgi) primer developing software (Table 1) (Untergasser et al. 2012). Preparation of amplicons for sequencing was conducted using the two-step conventional PCR. Reverse transcription was performed in a thermal cycler (Veriti™ 96-Well Thermal Cycler, Applied Biosystems, Foster City, CA) High Capacity cDNA Reverse Transcription Kit with RNase Inhibitor (Applied Biosystems, Foster City, CA) combined with Anchored Oligo (dT)_20_ Primer (Thermo Scientific, Waltham, MA) using 1 µl of Anchored Oligo dT Primer, 2 μl of 10× RT Buffer, 0.8 μl of 25× dNTP Mix (100 mM), 2 μl of 10× RT Random Primers, 1 μl of 50 U/μl MultiScribe™ Reverse Transcriptase, 1 μl of RNase Inhibitor (Applied Biosystems), 3.2 μl of molecular grade water, and 9 μl of template RNA. Cycling conditions were as follows: 37°C for 60 min and 95°C for 5 min. Amplification of complementary DNA (cDNA) was performed in a thermal cycler (Veriti™ 96-Well Thermal Cycler, Applied Biosystems, Foster City, CA) using 25 μl of master mix (Q5 High Fidelity 2× Master Mix, New England Bio Labs, Ipswich, MA), 0.25 μl of cDNA template, and 500 nM of each forward and reverse primers in a final volume of 50 µl. Cycling conditions were as follows: 98°C for 30 s, thirty-five cycles consisting of three steps (98°C for 10 s; 64°C, 65°C, or 67°C depending on primer set for 30 s; and 72°C for 30 s), and 72°C for 2 min.

**Table 1.** Sequences and combinations of primers used for the conventional reverse transcription PCR for sequencing of the EVEV E1 gene.

Primer name	Genome position (bp)		Sequence
Forward 9796	9796		GAGTCCTTGGACCACCTCTG
Forward 10279	10279		AAATCAGAGGATTGCCTTGC
Forward 10804	10804		GCCTTGTTTACCAGGGTGTC
Reverse 10657	10657		CCTGAGTGTATGGCACATGG
Reverse 11140	11140		TTTGGGCATGATACTGTGGA
Reverse 11327	11327		CAATCGCCGCAAGTTCTATC
Set name	Forward primer	Reverse primer	Cycling temperature for step 2-7
Set 2	Forward 9796	Reverse 10657	67°C
Set 4	Forward 10279	Reverse 11140	64°C
Set 6	Forward 10804	Reverse 11327	65°C
Set 7	Forward 9796	Reverse 11327	65°C

Direct purification of amplicon cDNA was performed using a commercial kit (PureLink™ Quick PCR Purification Kit, Thermo Scientific, Waltham, MA). Sanger sequencing was performed on the purified cDNA by a commercial firm (Eurofins, Louisville, KY). Contiguous DNA segments from each sample were assembled to form a complete E1 gene sequence using sequence analysis software (Sequencher^®^, Gene Codes Corporation, Ann Arbor, MI). Sequences have been deposited in GenBank with accession numbers MZ579546–MZ579560.

### Phylogenetic and phylogeography analyses

Our data set included the E1 gene sequences of two previous samples obtained in 1963 from Mahogany Hammock in the Everglades National Park (Chamberlain et al. 1969) (NCBI genome accession number AF075251) and in 2013 from the Everglades National Park (NCBI KR260737.1), for which the full genome was previously obtained and publicly available online. The data set was utilized to perform maximum likelihood (ML) phylogenetic tree using IQ-TREE using the best-fitting nucleotide substitution model according to the Bayesian information criterion (Trifinopoulos et al. 2016; Nguyen, von Haeseler, and Minh 2018) and an ultrafast bootstrap approximation (1,000 replicates) to assess the robustness of the phylogeny internal branches (Minh, Nguyen, and von Haeseler 2013). We assessed the quality of the phylogenetic signal present in the data sets by using IQ-TREE (Schmidt et al. 2002; Trifinopoulos et al. 2016; Nguyen, von Haeseler, and Minh 2018) and TempEst (Rambaut et al. 2016) to check, respectively, for presence of phylogenetic (Supplementary Fig. S1A) and temporal signals (Supplementary Fig. S1B). Molecular clock calibration for time of the most common ancestor (TMRCA) estimation was performed using the ML phylodynamic framework and the AF075251_EVEV_Fe3-7c_1963 as an outgroup in TreeTime (Sagulenko, Puller, and Neher 2018) (https://github.com/neherlab/treetime). TreeTime estimated a mean evolutionary rate of 1.73 · 10^−4^ nucleotide substitutions per site per year (n/s/y). A posterior distribution of EVEV phylogenies was inferred with the Bayesian phylodynamic framework implemented in BEAST v1.10.4 by enforcing the HKY substitution model, a strict molecular clock assuming the evolutionary rate estimated with TreeTime as a prior (1.73 · 10^−4^ n/s/y), and a constant demographic prior. The reconstruction of ancestral locations (Everglades, Big Cypress, and Fakahatchee) as discrete traits along the trees was performed by using the asymmetric transition model implemented in BEAST. A Markov chain Monte Carlo was run for 200 million generations, sampling every 20,000 generations. Proper mixing of the Markov chain was assessed by calculating the effective sampling size of each parameter estimate, which resulted in >200 for all parameters at the end of the run, after 10 per cent burn-in. Finally, a maximum clade credibility (MCC) tree was calculated, for each data set, from the posterior distribution with TreeAnnotator in the BEAST package. Phylogenetic trees were analyzed and graphically edited in FigTree v1.4.2 (http://tree.bio.ed.ac.uk/software/figtree/).

### Structural analyses

Due to the absence of a resolved EVEV structure, a template of VEEV (PDB ID: 3J0C) was used for mutations’ mapping, based on Blastp results (Query Cover = 100 per cent; *E*-value = 0.0; Percent identity = 96.61 %) against the Protein Data Bank (https://www.rcsb.org/) using reference sequence AF075251_EVEV_Fe3-7c_1963, as a query. Visualization of the atomic model is made with Chimera v1.12 (Pettersen et al. 2004).

## Results

PCR analysis of the *C. cedecei* pooled samples collected (*n *= 583) over the 8-week period in September and October 2017 showed that 19 samples (3.25 per cent) were positive for EVEV, as detected by PCR. Sequencing was successfully conducted on fifteen of the nineteen positive samples. Four samples were excluded due to low RNA concentration. All fifteen sequenced samples were confirmed to be EVEV with 99 per cent average percent identity match to the reference strain (accession number KR260737). The nucleotide base substitutions were aligned using GenBank KR260733 and AF075251 isolated from the Everglades National Park in 2013 and 1963, respectively, as reference sequences (Supplementary Table S1) (Kinney et al. 1998; Forrester et al. 2017). Ten non-synonymous mutations were detected across the fifteen samples with a base substitution frequency of 0.00676 ± 0.00676 (SE = 0.00178). For the analysis of amino acid composition, nucleotide positions were based on the E1 gene from reference sequence KR260737, and ten non-synonymous mutations were detected (Supplementary Table S2). The A116V and H441R mutations are shared among all of the isolates compared to the reference strain first isolated in the Mahogany Hammock in the Everglades National Park (Chamberlain et al. 1964). The A395T mutation was present in all of the Big Cypress isolates as well as in a single Fakahatchee isolate (301_Fakahatchee_G8_9.6.17).

**Figure 2.**
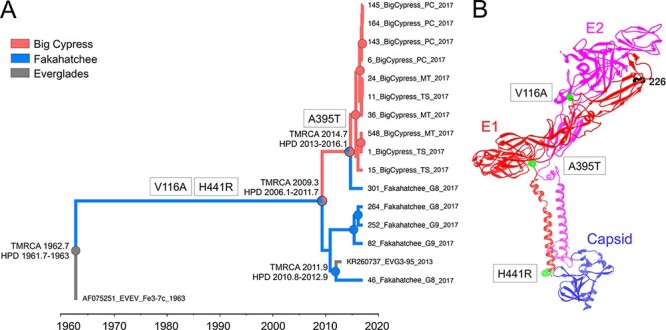
Spatiotemporal spread of EVEV in the Greater Everglades and position of unique mutations on E1. (A) MCC time-scaled phylogeny inferred by enforcing a strict clock and constant demographic prior in BEAST 1.10.4. Time of coalescent events (TMRCA) and 95 per cent HPD intervals (HPD) at major divergent event nodes are reported near nodes. The pie chart, at nodes for which the posterior probability was greater than 0.9, shows the ancestral state reconstruction probability for the origin. Branch lengths are scaled in time and colored according to location based on the legend in the figure. Mutations of relevance that number is the amino acid position of the protein where the mutation was mapped in the tree. (B) Mutations of interest are shown by green spheres on the E1 protein using the structural data available for the Capsid, E1, and E2 structure of the VEEV TC-83 strain (PDB ID: 3J0C). The black sphere indicated Position 226, site of the A226V mutation in E1 that allowed CHIKV to expand the host range.

Next, we performed a Bayesian phylogeographic analysis to determine the phylogenetic relationship between the sequences obtained in the Big Cypress and Fakahatchee locations, the probable movement across these locations, and the time of their emergence. At the base of the MCC tree, we find the isolate Fe3-7c_1963, which was collected in 1963 in the Everglades National Park (Chamberlain et al. 1964) (Fig. 2A). The isolates obtained in the Big Cypress and Fakahatchee locations showed high compartmentalization, clustering in two clades that diverged in April 2009, TMRCA with 95 per cent high posterior density (HPD) interval of February 2006 and September 2011. The first clade is composed mainly of sequences obtained from Fakahatchee, and its emergence is dated between April 2009 and November 2011 (TMRCA for the clade, with HPD interval of October 2010–November 2012). The viral population sampled in Fakahatchee included the isolate KR260737_EVG3-95_7.2 obtained in 2013, suggesting a potential introduction event from Fakahatchee to the Everglades National Park (Forrester et al. 2017). The second clade is composed of sequences collected from Big Cypress that emerged sometime between the divergence event in April 2009 and September 2014 (this date being the TMRCA for the clade, with HPD interval of January 2013–February 2016). One viral isolate obtained in Fakahatchee (isolate 301_Fakahatchee_G8_2017) was found at the base of the Big Cypress subclade, suggesting that the EVEV population in Big Cypress moved to Fakahatchee. The mutational pattern along internal branches of the phylogeny at major divergence events also supports this hypothesis in that the A395T mutation is specific to the Big Cypress viral population and the one isolate collected in Fakahatchee (Fig. 2A). This mutation lies between Domain III and the transmembrane domain of the protein (Fig. 2B). The other two mutations of interest, A116V and H441R, were shared among all isolates obtained after its first isolation in 1963 and occurred in Domain II of E1 (A116V) and the transmembrane helix (H441R) (Fig. 2B). The A116V mutation is in the opposite position of the A226V mutation of Chikungunya virus (CHIKV) and, in addition to the K211E mutation, considered essential changes that allowed CHIKV to successfully infect *Aedes albopictus*, leading to the large outbreak in Réunion Island in 2005 and the subsequent expansion worldwide (Schuffenecker et al. 2006; Tsetsarkin et al. 2007).

## Discussion

The goal of this study was to investigate the evolution of EVEV, an enzootic pathogen endemic to Florida isolated from mosquitoes and wild rodents from the hardwood hammocks of the Everglades and Big Cypress ecosystems (Chamberlain et al. 1964, 1969), based on the E1 glycoprotein, a key player in cell fusion (Kielian, Chanel-Vos, and Liao 2010; Forrester et al. 2017). A total of 583 samples were collected from two sites in the Greater Big Cypress region consisting of Big Cypress and Fakahatchee Strand Preserve State Park. Nineteen of the samples were positive for EVEV by PCR, and the entire E1 protein gene was successfully sequenced from fifteen of these samples, allowing for phylogenetic and structural analyses. Based on the available reference strains, we detected ten non-synonymous mutations in the E1 protein. Through our analysis, compartmentalization was demonstrated based on the location of sampling indicating two monophyletic subclades that diverged in 2009. Three mutations reside within the E1 glycoprotein consisting of A116A, H441R, and A226V. Two of these mutations were shared by all EVEV samples except the 1963 reference strain, and one was specific to Big Cypress. Without functional studies, the importance of these mutations can only be speculative; however, these few changes in the E1 protein may be very important in the light of the recent introduction of a suspected vector for VEEV, *C. panocossa* into Florida. Single mutations have been responsible for changes in vector specificity in alphaviruses resulting in new or intensification of outbreaks. In 2005/6, the mutation in the E1 of CHIKV, A226V, was responsible for greater adaptation to *A. albopictus* and is considered responsible for the intensity of the epidemic in the islands of the Indian Ocean (Schuffenecker et al. 2006; Tsetsarkin et al. 2007). In 2009, another mutant arose with a single mutation in the E1 protein and another in the E2 protein conferring increased viral fitness of CHIKV to *A. Albopictus*, which contributed substantially to epidemics in India (Niyas et al. 2010; Agarwal et al. 2016; Tsetsarkin and Weaver 2011). The H441R mutation resides within the E1 transmembrane helix, near the capsid protein, which occurred in all of the strains following the 2013 strain. The A395T mutation appears to be specific to Big Cypress and is found between Domain III and the transmembrane domain of the protein. Mutations in Domain III result in loss of pH-dependent fusion reactions as demonstrated for VEEV (Lescar et al. 2001; Roussel et al. 2006). Nonetheless, comparisons between different complexes of alphaviruses may have limited significance. The host cell receptor for CHIKV and other arthritogenic alphaviruses is MXRA8 (Zhang et al. 2018), while LDLRAD3-D1 has recently been shown to be the receptor for VEE (Basore et al. 2021; Ma et al. 2020; 2021). Future studies on viral cell entry, tropism, and replication will help to clarify the functional repercussions of the mutations seen here.

EVEV is compartmentalized by location, Big Cypress and Fakahatchee, and diverged into two monophyletic clades in 2009, reflecting likely multiple movements of EVEV across the Everglades: to Fakahatchee, from here to the Everglades National Park, and from Big Cypress to Fakahatchee. However, more genomic analyses from sampling in the Everglades National Park to understand clearly the circulation and the directionality of EVEV. The A395T mutation occurs solely within the Big Cypress viral population, but one isolate collected in Fakahatchee also presents this mutation indicating the current movement of EVEV across regions.

The similarity of strains from Fakahatchee and the Everglades National Park is surprising, given that Big Cypress effectively lies between these two wetland areas. The isolates from Big Cypress and Fakahatchee diverged following Hurricane Andrew in Florida, the most destructive hurricane to ever hit Florida, causing billions of dollars in damage (Willoughby and Black 1996). Hurricane Andrew moved into South Florida, passing from the Everglades National Park to the Big Cypress Preserve with an accompanying profound change in water levels (Orr 1992). While causation cannot be established with available samples, the severe climatic event may have driven the dispersal of these two variants between these geographically distinct sites.

Water levels may be the primary effect of weather and climate upheaval. A comprehensive study of mosquito-borne viruses isolated from mosquitoes trapped in the Greater Everglades was recently published in which over 350,000 female mosquitoes were collected in 2014–5 and 2,010 mosquito pools were cultured for arboviruses (Fish et al. 2021). In terms of abundance, *C. cedecei* was the fourth highest of thirty mosquito species collected during the high water year. Sixteen isolates of EVEV were recovered resulting in a pooled positivity rate of 2.75 per cent. While this pool-positivity rate is slightly lower than in our current study, there were 105 collection sites in this recent publication, and EVEV was recovered from 14 of these sites. During periods of high water in the Everglades, EVEV was the most common virus isolated, followed by Keystone virus, Shark River virus, Mahogany Hammock virus, Gumbo Limbo virus, and St. Louis encephalitis virus. Tensaw virus, EVEV, and Shark River virus were also isolated during low water-level periods. EVEV was isolated from other species of mosquitoes including seven pools of *C. nigripalpus* and one to two pools of *A*. *atlanticus, C*. *atratus, C*. *Quiquefasciatus*, and *Wyeomyia mitchellii*.

Our study detected a relatively high percentage of EVEV-positive *C. cedecei* mosquito pools (3.25 per cent). This stands in contrast to the findings of investigations in the 1960s in Big Cypress, where over 200,000 mosquitoes were collected (including nearly 8,000 *Culex* (*Melanoconion*) species), but no EVEV was isolated (Chamberlain et al. 1969). The cotton rats and cotton mice trapped in Big Cypress in the 1970s were rarely seropositive for EVEV, whereas a third or more of cotton rats and mice trapped in the Everglades National Park were seropositive (Lord et al. 1973). An early serosurvey of members of the Seminole tribe in Big Cypress in 1960 demonstrated very high EVEV seropositivity in adults 84 per cent of individuals aged ≥16 years, but children under 8 years of age were seronegative, suggesting that EVEV transmission likely waxed and waned in Big Cypress (Work 1964). Thus, it was theorized that Big Cypress is a ‘sink’ habitat, where EVEV spills over periodically from the Everglades National Park (the ‘source’). The high percentage of infected mosquito pools in our study may therefore indicate that sampling occurred during such a period of expanded transmission, or this may represent a sustained increase in transmission owing to reduced mammal diversity and focused feeding upon rodents (Burkett-Cadena et al. 2021).

In light of evidence that a principal mode of RNA viral macroevolution is host-switching (Kitchen, Shackelton, and Holmes 2011), the mutations we have found here possibly reflect changes in host usage after the dramatic reduction in mammal biodiversity following the introduction, establishment, and spread of the invasive Burmese python in the 1990s (Dorcas et al. 2012). In prior studies on EVEV in the Greater Everglades, we describe how the composition of hosts that the vector feeds on has changed over time (Wu, Sklar, and Rutchey 1997; Wu, Wang, and Rutchey 2006; Burkett-Cadena et al. 2021). The cotton rat has become the main source of blood meals, reflecting a reduction in biodiversity that correlates with the establishment of the Burmese python.

Increased surveillance and study of EVEV are warranted. The regions abutting Big Cypress have undergone significant land-use change and population growth. Agricultural fields (predominantly citrus and vegetable crop, to a lesser degree sugarcane, cattle, and ornamental) are adjacent to the Fakahatchee Strand Preserve State Park (Harwell 1998). Farmland acreage has halved between 1997 and 2012, while urbanization expanding from coastal areas has increased the population by 17 per cent between 2010 and 2019. Thus, mutations in the envelope protein coupled with loss and fragmentation of habitat increase the likelihood of human and animal infections in South Florida. This is especially true now, given that new areas of contact and spillover to the human population may be arising from urbanization, ecological changes in the Everglades, and rising sea levels in South Florida (Ingebritsen). Studying the complex relationships between these elements and their influence on viral evolution will have broader applications as well, furthering our understanding of arboviral and zoonotic disease ecology. Whole genome sequencing of these and additional isolates, as well as refinement of phylodynamics coupled with spatiotemporal analyses, is required to further resolve the impact of the changing ecology in the Greater Everglades. Ultimately both *in vivo* and *in vitro* studies are necessary to fully investigate any changes in infectivity associated with these and other mutations associated with these analyses.

## Supplementary Material

veac111_Supp
