# Supplementary material for: Everglades virus evolution: Genome sequence analysis of the envelope 1 protein reveals recent mutation and divergence in South Florida wetlands
Source: Virus Evol. 2022 Dec 14;8(2):veac111. doi: 10.1093/ve/veac111 (PMC9795574; doi:10.1093/ve/veac111)
Supplement: veac111_Supp [file veac111_supp.zip › supplementary material.docx]

**Supplementary material**

**Figure S1. Phylogenetic quality of the EVEV data sets. (A)** Presence of phylogenetic signal was evaluated by likelihood mapping checking for alternative topologies (tips), unresolved quartets (center) and partly resolved quartets (edges) for the data set. **(B)** Linear regression of root-to-tip genetic distance within the ML phylogeny against sampling time for each taxon: temporal resolution was assessed using the slope of the regression, with positive slope indicating sufficient temporal signal.

**Table S1. Nucleotide changes detected in the Everglades virus E1 gene between historical Genbank sequences and sequences from 15 mosquito pools collected from south Florida wildlife refuges in 2017.**

| **Nucleotide Position** | **25** | **27** | **33** | **72** | **90** | **99** | **138** | **159** | **230** | **347** | **371** | **375** | **384** | **392** | **393** | **447** | **492** | **540** | **606** | **759** | **786** | **877** | **888** | **912** | **950** | **1020** | **1025** | **1095** | **1122** | **1170** | **1183** | **1269** | **1322** |
| --- | --- | --- | --- | --- | --- | --- | --- | --- | --- | --- | --- | --- | --- | --- | --- | --- | --- | --- | --- | --- | --- | --- | --- | --- | --- | --- | --- | --- | --- | --- | --- | --- | --- |
| **KR260737_EVG3-95_2013** | **A** | **C** | **C** | **T** | **C** | **G** | **C** | **G** | **A** | **T** | **C** | **T** | **G** | **C** | **G** | **T** | **C** | **C** | **T** | **T** | **C** | ***A** | **C** | **C** | **C** | **G** | **C** | **T** | **C** | **C** | **G** | **T** | **G** |
| **AF075251_EVEV_Fe3-7c_1963** | **.** | **T** | **.** | **.** | **.** | **A** | **.** | **A** | **.** | **C*** | **.** | **C** | **A** | **.** | **.** | **.** | **T** | **.** | **C** | **.** | **.** | **.** | **.** | **.** | **.** | **A** | **.** | **C** | **T** | **T** | **.** | **.** | ***A** |
| **6_BigCypress_PC_9.5.17** | G* | . | T | C | . | A | . | . | . | . | . | C | A | . | . | C | T | . | C | C | . | . | . | T | . | . | . | . | . | T | *A | . | . |
| **1_BigCypress_TS_9.5.17** | . | . | T | . | . | A | . | . | G* | . | . | C | A | . | . | C | T | . | C | C | . | . | . | . | . | . | . | . | . | T | *A | . | . |
| **11_BigCypress_TS_9.5.17** | . | . | T | . | . | A | . | . | . | . | . | C | A | . | . | C | T | . | C | C | . | . | . | T | . | . | . | . | . | T | *A | . | . |
| **15_BigCypress_TS_9.5.17** | . | . | T | . | . | A | T | . | . | . | . | C | A | *T | . | C | T | . | C | C | . | . | . | . | . | . | . | . | . | T | *A | . | . |
| **24_BigCypress_MT_9.5.17** | . | . | T | . | . | A | . | . | . | . | . | C | A | . | . | C | T | . | C | C | . | . | . | T | . | . | . | . | . | T | *A | . | . |
| **36_BigCypress_MT_9.5.17** | . | . | T | . | . | A | . | . | . | . | . | C | A | . | . | C | T | . | C | C | . | . | . | T | . | . | . | . | . | T | *A | . | . |
| **46_Fakahatchee_G8_9.5.17** | . | . | . | . | . | A | . | . | . | . | . | C | . | . | . | . | T | T | . | . | . | . | T | . | . | . | . | . | . | . | . | . | . |
| **82_Fakahatchee_G9_9.6.17** | . | . | . | . | . | A | . | . | . | . | . | C | A | . | . | . | T | . | C | . | . | . | . | . | . | . | . | . | . | . | . | C | . |
| **143_BigCypress_PC_9.5.17** | . | . | T | C | . | A | . | . | . | . | . | C | A | . | . | C | T | . | C | C | . | . | . | T | . | . | . | . | . | T | *A | . | . |
| **145_BigCypress_PC_9.5.17** | . | . | T | C | . | A | . | . | . | . | . | C | A | . | . | C | T | . | C | C | . | . | . | T | . | . | . | . | . | T | *A | . | . |
| **164_BigCypress_PC_9.5.17** | . | . | T | C | . | A | . | . | . | . | . | C | A | . | . | C | T | . | C | C | . | . | . | T | . | . | . | . | . | T | *A | . | . |
| **252_Fakahatchee_G9_9.6.17** | . | T | . | . | . | A | . | . | . | . | . | C | A | . | A | . | T | . | C | . | T | . | . | . | *T | . | . | . | . | . | . | C | . |
| **264_Fakahatchee_G8_9.6.17** | . | T | . | . | . | A | . | . | . | . | . | C | A | . | . | . | T | . | C | . | . | . | . | . | . | . | *T | . | . | . | . | C | . |
| **301_Fakahatchee_G8_9.6.17** | . | . | T | . | T | A | . | . | . | . | . | C | A | . | . | C | T | . | C | . | . | *T | . | . | . | . | . | . | . | T | *A | . | . |
| **548_BigCypress_MT_10.25.17** | . | . | T | . | . | A | . | . | G* | . | T* | C | A | . | . | C | T | . | C | C | . | . | . | . | . | . | . | . | . | T | *A | . | . |

**Table S2. Amino acid comparison of non-synonymous mutations in Everglades virus E1 protein between 15 samples collected from south Florida wildlife parks in 2017.**

|  | | | | | | | | |  |  |  |
| --- | --- | --- | --- | --- | --- | --- | --- | --- | --- | --- | --- |
| **Amino Acid Position (residue number)** | **9** | **77** | **116** | **124** | **131** | **293** | **317** | **342** | | **395** | **441** |
| KR260737_EVG3-95_2013 | N | Q | V | A | A | T | T | A | | A | R |
| AF075251_EVEV_Fe3-7c_1963 | . | . | A | . | . | . | . | . | | . | H |
| 6_BigCypress_PC_9.5.17 | D | . | . | . | . | . | . | . | | T | . |
| 1_BigCypress_TS_9.5.17 | . | R | . | . | . | . | . | . | | T | . |
| 11_BigCypress_TS_9.5.17 | . | . | . | . | . | . | . | . | | T | . |
| 15_BigCypress_TS_9.5.17 | . | . | . | . | V | . | . | . | | T | . |
| 24_BigCypress_MT_9.5.17 | . | . | . | . | . | . | . | . | | T | . |
| 36_BigCypress_MT_9.5.17 | . | . | . | . | . | . | . | . | | T | . |
| 46_Fakahatchee_G8_9.5.17 | . | . | . | . | . | . | . | . | | . | . |
| 82_Fakahatchee_G9_9.6.17 | . | . | . | . | . | . | . | . | | . | . |
| 143_BigCypress_PC_9.5.17 | . | . | . | . | . | . | . | . | | T | . |
| 145_BigCypress_PC_9.5.17 | . | . | . | . | . | . | . | . | | T | . |
| 164_BigCypress_PC_9.5.17 | . | . | . | . | . | . | . | . | | T | . |
| 252_Fakahatchee_G9_9.6.17 | . | . | . | . | . | . | I | . | | . | . |
| 264_Fakahatchee_G8_9.6.17 | . | . | . | . | . | . | . | V | | . | . |
| 301_Fakahatchee_G8_9.6.17 | . | . | . | . | . | S | . | . | | T | . |
| 548_BigCypress_MT_10.25.17 | . | R | . | V | . | . | . | . | | T | . |
